# The safety of neoadjuvant chemotherapy combined with non-tube nofasting fast-track surgery for esophageal carcinoma

**DOI:** 10.3389/fonc.2022.906439

**Published:** 2022-08-31

**Authors:** Yan Zheng, Wentao Hao, Yin Li, Xianben Liu, Zongfei Wang, Haibo Sun, Shilei Liu, Wenqun Xing

**Affiliations:** ^1^ Department of Thoracic Surgery, The Affiliated Cancer Hospital of Zhengzhou University, Henan Cancer Hospital, Zhengzhou, China; ^2^ Department of Thoracic Surgery, National Cancer Center/National Clinical Research Center for Cancer/Cancer Hospital, Chinese Academy of Medical Sciences and Peking Union Medical College, Beijing, China

**Keywords:** esophageal cancer, fast track surgery, neoadjuant chemotherapy, minimally invasive esophagectomy, short term outcomes

## Abstract

Our non-tube no fasting (early oral feeding and no nasogastric tube) fast-track surgery (FTS) was safe and effective to combine with neoadjuvant chemotherapy for McKeown minimally invasive esophagectomy. In addition, the two groups were similar in terms of the recovery time, hospital discharge day, and early resumption of oral feeding.

**Objectives:**

To evaluate the safety of early oral feeding (EOF) combined with neoadjuvant chemotherapy (NAC) of esophagectomy.

**Summary Background Data:**

Our non-tube no fasting (early oral feeding and no nasogastric tube) fast-track surgery (FTS) was safe and effective for primary surgery esophageal cancer patients.

**Methods:**

We retrospectively evaluated consecutive patients who underwent non-tube no fasting and McKeown minimally invasive (MIE). They were divided into two groups: one received NAC, and the other received primary surgery. Complications after the operation, postoperative CRG complications, operative time, operative bleeding, and length of stay were evaluated.

**Results:**

Between 01/2014 and 12/2017, there hundred and eighty two consecutive patients underwent MIE with total two-field lymphadenectomy under the non-tube no fasting fast-track surgery program. A total of 137 patients received NAC, and 245 accepted primary surgery. Propensity score matching was used to compare NAC patients with 62 matched patients from each group. The NAC group had a similar number of total complications as the primary surgery group (32.26% in the primary surgery group vs. 25.81% in the NAC group; p=0.429) and had the same median postoperative hospitalization duration (8 days, p=0.723).

**Conclusions:**

After McKeown MIE, the patients receiving NAC combined with “non-tube no fasting” FTS had a similar incidence of postoperative complications outcomes as those without NAC. In addition, the two groups were similar in terms of the recovery time, hospital discharge day, and early resumption of oral feeding.

## Introduction

Esophageal cancer has a high incidence rate in China. Surgical treatment is the primary method to cure local advanced resectable esophageal cancer. However, it presents high morbidity and mortality, even in high-volume centers. The National Oesophago-Gastric Cancer Audit reported a morbidity rate of 3.2% and mortality rate of 29.7% for esophagectomy (1). If the patient has anastomotic leakage, the posthospital stay can be increased up to 43 days (1).

To reduce the morbidity, mortality and hospitalization duration of these patients, the concept of fast-track protocols after surgery was initially introduced by Kehlet in 1997 (2). It was soon successfully adopted in gastric and colon surgery (3). However, it was difficult to introduce to esophagectomy. Fasting prohibited fast-track surgery (FTS) in esophagectomy. Seven years after the initial FTS concept, it was introduced to esophagectomy by Cerfolio et al. (4) In 2011, our team reported the first application of early oral feeding and non-nasogastric tube (non-tube no fasting) FTS for esophagectomy (5). It soon caused considerable controversial in fear of anastomotic leakage. However, the most difficult aspect was combining this approach with preoperative treatment. In the review by Gemmill, all 10 studies excluded patients undergoing neoadjuvant treatment, which may increase anastomotic leakage (6).

More than ten years have passed since the first attempt of “non-tube no fasting” FTS after esophagectomy. We also wanted to confirm its safety and feasibility in combination with NAC for MIE. The short term outcomes of neoadjuvant chemotherapy (NAC) and primary surgery were compared for EC patients with “non-tube no fasting” FTS.

## Methods

### General information

The study was approved by the Ethics Review Committee of the Affiliated Cancer Hospital of ZhengZhou University/Henan Cancer Hospital (number 2016ct081).

In this study, the inclusion criteria were as followed: 1.consecutive patients ESCC patient who underwent surgery between 3 January 2014 and 29 December 2017. 2. with R0 resected ESCC. 3.Surgery was performed in the strict one of the thoracic surgery department of Henan Cancer Hospital. Exclusion criteria: 1.Patients who remained in the intensive care unit (ICU) for more than 1 day. 2.Patients with bilateral recurrent laryngeal nerve (RLN) injury. Preoperative tests included enhanced abdominal and cervical color ultrasound, thoracic and upper abdominal computed tomography (CT) scanning, endoscopic ultrasound (EUS), pathological examination, emission computed tomography (ECT) and other routine examinations.

### Surgical procedures

All patients underwent MIE surgical approaches, as previously described (7, 8). Briefly, the left lateral decubitus position was adopted, and four ports were inserted into the thoracic cavity. The azygous vein was divided, and the esophagus was mobilized. The right and left recurrent laryngeal nerve and subcarinal and lower mediastinal nodes were harvested. For the abdominal part, the patient was placed in the supine position, and five ports were inserted into the abdominal cavity. The stomach was mobilized, and a gastric conduit was made by using linear staplers (EC60, Ethicon, Cincinnati, OH, USA). The left gastric artery, common hepatic artery, and splenic lymph nodes were removed en bloc. A hand-sewn cervical anastomosis approach was adopted for esophagogastric anastomosis on the left side of the neck (9). The thoracic duct was preserved normally. A chest drainage was put in thoracic and abdominal cavity (10).

### Follow-up

During the first 2 years, the patients visited our patient department or were followed up by phone every 3 months. From the third year to the fifth year, follow-up occurred every six months, and from the sixth year, follow-up occurred annually. Follow-up examinations included chest CT scans and abdominal and cervical ultrasound. Other examinations were performed based on the patient’s symptoms. The date from surgery to the first date of neoadjuvant treatment was defined as overall survival (OS). May 3, 2020, was the last follow-up date. Not all the patients did their follow up in out patient department. Some of the patients were follow-up by research nurse of our department by phone and all of them were follow-up by LinkDoc company for our hospital.

### Statistical analysis

The Mann–Whitney U test and the chi-square test were adopted to compare the clinicopathological qualitative variables between the two groups. Student’s t test was used for quantitative data, and Fisher’s exact test was used for categorical variables. IBM SPSS statistics version 23 (IBM Corporation, Armonk, NY, USA) was employed for statistical analysis. A p value/0.05 was considered statistically significant. To reduce the bias between the two groups, propensity score (PS)-matched analysis was adopted. The matched variables included age, sex, BMI, clinical TNM stage, history of disease, surgical time, bleeding volume during surgery, and performance status score.

### EOF group

On the morning of the first day after the operation, the patient was allowed to sip liquid. If the patients had no symptoms of nausea, vomiting or aspiration. Then the patients could start to consume food at will after fifty chews for every bite of food before swallowing (11). This was monitored by nurse for the first time and then by caretaker.

The basic nutrition for EOF patients was parenteral nutrition, including glucose, amino acids and fat emulsion, which offered 1000 to 1500, 800 to 1000, and 500 to 800 kilocalories (kcal) on POD1, POD2, and POD3, respectively. Oral feeding was started on POD1. The Harris-Benedict formula was used to calculate the required caloric intake of each patient by dieticians. Nutrition education was provided by dieticians. The nurse would emphasize the need for strict aspiration precautions. On POD1, more liquid diet was encouraged, such as porridge, milk, and juice. Semiliquid foods and soft solid foods were provided from POD2, such as cakes, boiled eggs, rice, steamed bread and noodles. The fifty chews per bite of food method was required to ensure patients chewed the food completely and that it had been transformed into a semiliquid state. Normally, parenteral nutrition is removed on POD4 or POD5.

### Traditional group

In the traditional group, nasogastric and nasoenteral feeding tubes were used. The patients received nutrition *via* a nasoenteral feeding tube from POD 1. Parenteral nutrition was also adopted. Normally, the nasogastric and nasoenteral feeding tube was removed on POD 7, and the patients resumed oral feeding under the guidance of dieticians.

## Results

**Figure 1:**
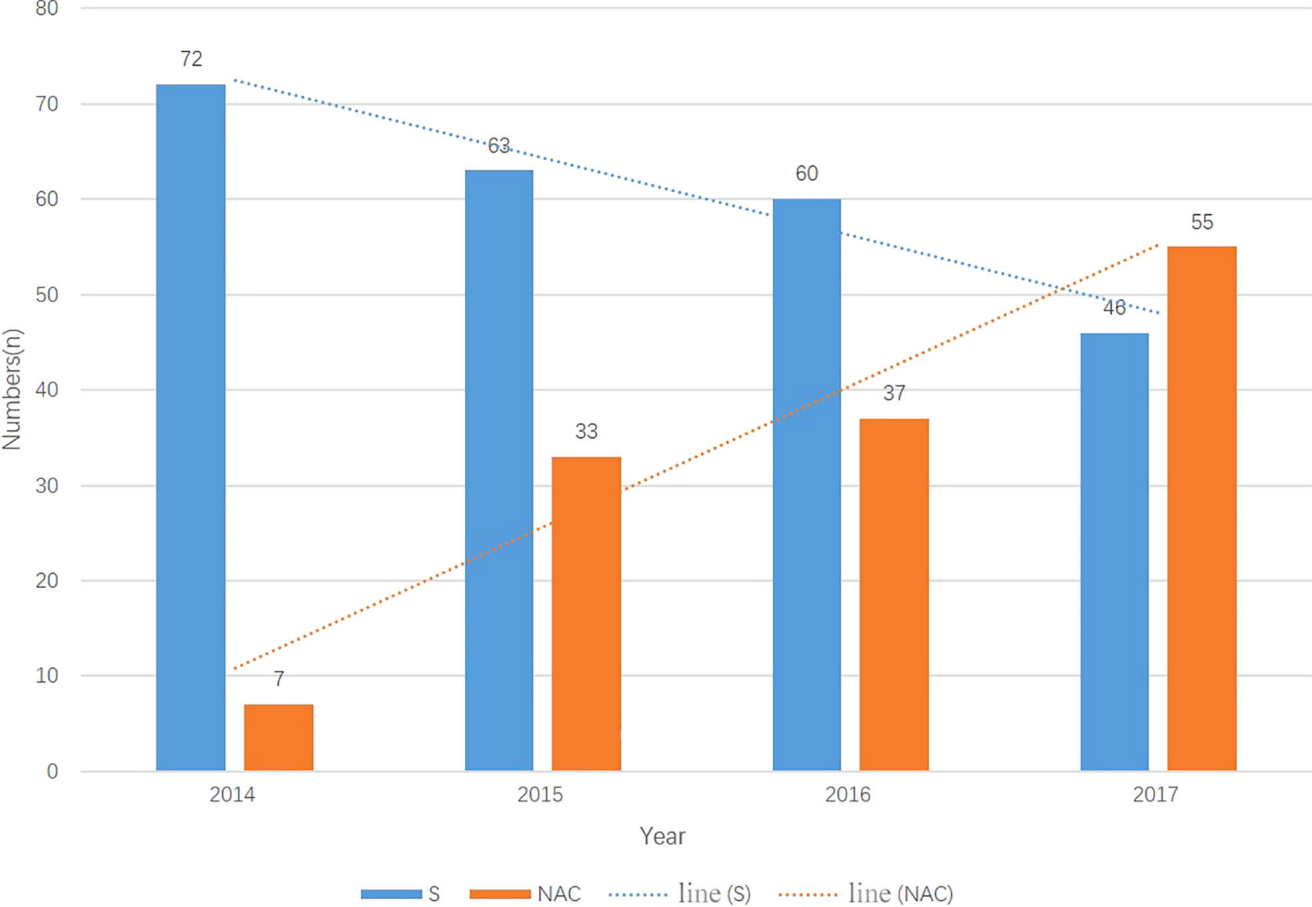
The number of patients in two groups each year from 2014-2017. The number of primary surgery patients droped every year meanwhile the muber of NAC patients increased every year. N, number; S, primary surgery; NAC, neoadjuvant chemotherapy.

**Figure 2:**
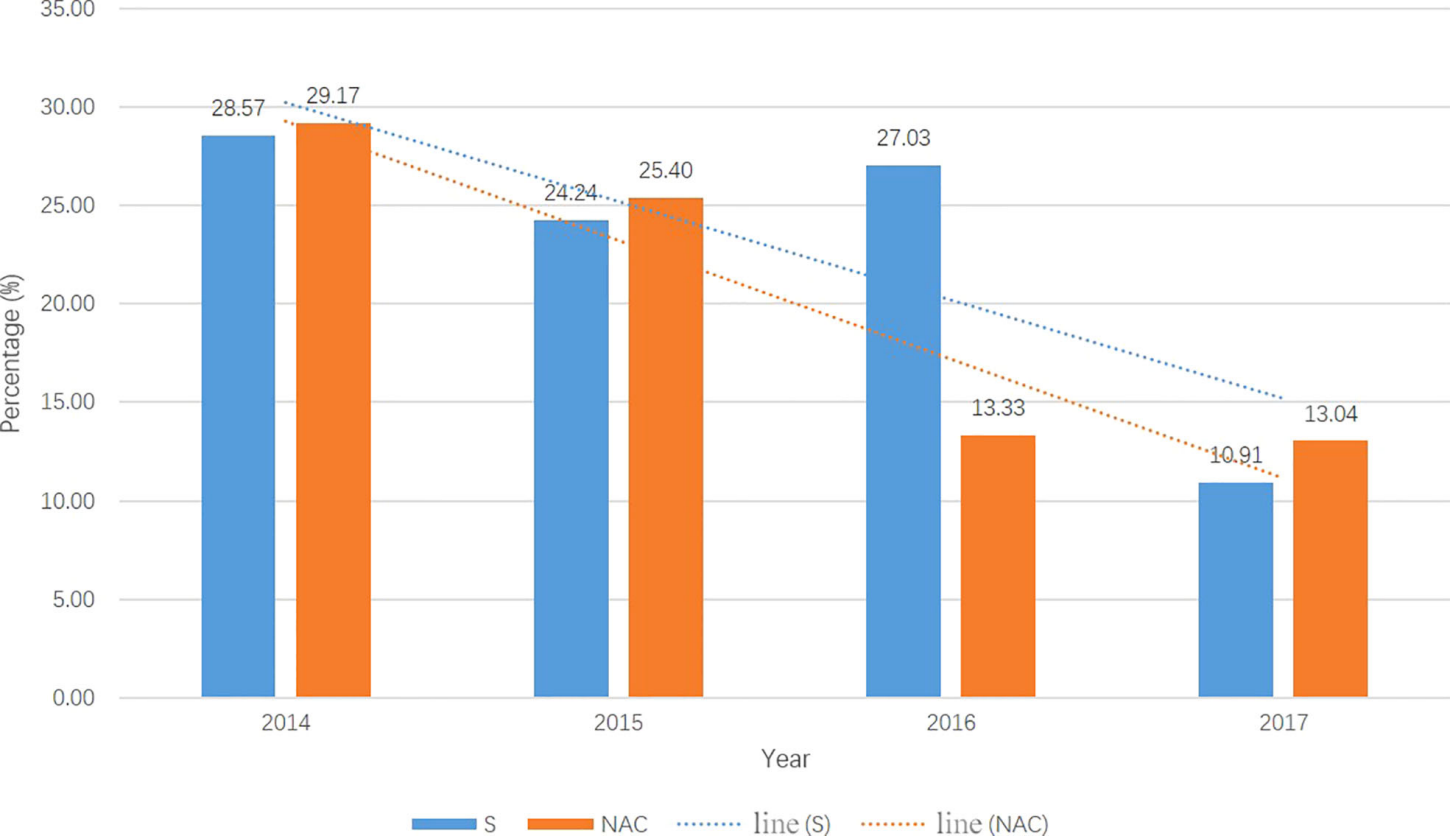
The total postoperation complication rate in two groups each year from 2014-2017. The total complication rates were droped year by year in both groups. S, primary surgery; NAC, neoadjuvant chemotherapy.

From 01/2014 to 12/2017, a total of 382 consecutive patients met the inclusion criteria; 137 patients received NAC, and 245 underwent primary surgery. Beginning in 2014, an increasing number of patients received NAC and underwent “non-tube no fasting” FTS (**Figure 1**). At the same time, the total complication rate in both groups declined year by year (**Figure 2**). A total of 124 matched patients were retained after PS matched analysis. Each group had 62 patients (**Table 1**). The baseline demographics of the 124 patients after PS matching analysis are summarized in **Table 1**. The primary clinical data were comparable. The patients in the primary surgery group had a slightly earlier pathological stage (P=0.077), and those in the NAC group were slightly younger (P=0.184).

**Table 1:** Baseline demograpic and clinical characteristics of esophageal carcinoma patients after PMS.

Variable	S (N = 62)	NAC (N = 62)	*χ2/F/W*	*P* value
**Mean Age(range)**	62.10 (45-78)	60.34 (37-72)	1.336	0.184
**Mean BMI(SD)**	23.42 (3.54)	24.22 (3.12)	-1.178	0.241
**Sex N(%)**			0.911	0.340
Male	39 (62.90)	44 (70.97)		
Female	23 (37.10)	18 (29.03)		
**History N(%)**			0.525	0.469
No	37 (59.68)	33 (53.23)		
Yes	25 (40.32)	29 (46.77)		
**cTNM stage N(%)**			5.139	0.077
I	11 (17.74)	9 (14.52)		
II	35 (56.45)	25 (40.32)		
III	16 (25.81)	28 (45.16)		
**Adjuvant treatment N(%)**			0.704	0.402
Yes	17 (27.42)	13 (20.97)		
No	45 (72.58)	49 (79.03)		

PMS, propensity matched score; S, surgery; NAC, neoadjuvant chemotherapy; SD, +/-; N, number; F, F-test(joint hypotheses test); W, Wilcoxon-test; cTNM, clinical tumor lymph nodes metastasis stage.

The intraoperative and postoperative outcomes and total complication rates are shown in **Table 2**. No deaths occurred in either of the groups in the hospital or at 90 days after surgery. The median postoperative hospital stay and most objective recovery data were not significantly different (median 8 days in both groups, p=0.723). The median number of chest tube drainage days was 6 in both groups (p=0.131). These FTS-related protocol were in the same manner as those in our previous RCT (5). The mean operation time was 200 min in the primary surgery group and 222.5 min in the NAC group (P < 0.001). The median number of lymph nodes retrieved in the primary surgery group was lower than that in the NAC group (9 fewer nodes, P < 0.001). The pathological data were compared between the two groups, showing a p value of 0.049 (**Table 2**).

**Table 2:** Intraoperative and Postoperative Outcome.

	S (N = 62)	NAC (N = 62)	χ2/F/W	P value
**Intraoperative data**				
Mean operative time(SD) (min)	200(130-310)	222.5(150-350)	1223.5	<0.001*
Thoracic duct ligation(%)	14(22.58)	11(17.74)	0.451	0.502
Mean blood loss(SD) (mL)	67.26(42.59)	77.645(43.14)	-1.349	0.180
Median lymph nodes retrieved (range) N	24(15-56)	33(15-64)	1191	<0.001*
**Postoperative data**				
Complication N(%)	20(32.26)	16(25.81)	0.626	0.429
Median postoperative hospital stay days (range)	8(6-94)	8(5-34)	1852.0	0.723
Median Chest tube drainage days (range)	6(4-93)	6(4-31)	1626.5	0.131
Readmission to ICU N (%)	2(3.23)	1(1.61)	NA	1.000
**Pathological data**				
**Type of carcinoma N(%)**			0.121	0.989
Squamous cell carcinoma	55(88.71)	54(87.10)		
Adenosquamous carcinoma	2(3.23)	2(3.23)		
Adenocarcinoma	4(6.45)	5(8.06)		
Small cell carcinoma	1(1.61)	1(1.61)		
Median positive lymph nodes retrieved (range) N	0(0-12)	0(0-14)	1849	0.683
**pTNM/ypTNM staging 8^th^ N(%)**				
pCR	NA	7(11.29)	NA	0.049*
I	17(27.42)	15(24.19)		
II	28(45.16)	26(41.94)		
III	17(27.42)	14(22.58)		

S, surgery; NAC, neoadjuvant chemotherapy; N, number; F, F-test(joint hypotheses test); W, Wilcoxon-test; SD, +/-; ICU, Intensive Care Unit; NA, Not Available; pTNM, pathological tumor/node/metastasis; *Statistically significant (p<0.05).

The primary surgery and NAC groups showed no significant differences in terms of CRG complications and other complications (**Table 3**). The rates of anastomotic leakage in the primary surgery group and NAC group were 0 and 1.61% (1/6), respectively, representing a difference of -1.61% (95% CI -5.3% to 8.59%). The rates of unilateral RLN injury in the primary surgery group and NAC group were 6.45% (4/62) and 0, respectively, representing a difference of 6.45% (95% CI -0.57 to 15.45). The most common complication was pneumonia, which occurred in 11.29% (7/62) of primary surgery patients and in 9.68% (6/62) of NAC patients, representing a difference of 1.61% (95% CI -9.79% to 13.07%). Moreover, no notable differences in Clavien–Dindo grade complications were observed between the 2 groups. Two patients (3.23%) in the primary surgery group and 1 patient (1.61%) in the NAC group required reoperation (difference of 1.61%; 95% CI -5.74% to 9.52%). These patients were determined to have Clavien–Dindo grade IIIb complications. In the two patients in the primary group, one experienced bleeding at the neck incision, and the other experienced bleeding in the chest cavity. The patient in the NAC group was suspected to have mechanical intestinal obstruction. However, after abdominal exploration, no remarkable findings were observed. He was finally diagnosed with several intestinal tympanites. The 3 patients recovered quickly after surgery.

**Table 3:** The postoerative complications in two groups.

Variable N (%)	S (N = 62)	NAC (N = 62)	Difference (95% CI)	χ2	P value
**Respiratory Complications (total)**	11 (17.74)	10 (16.13)	1.61 (-11.78-14.97)	0.057	0.811
Pneumonia	7 (11.29)	6 (9.68)	1.61 (-9.79-13.07)	0.086	0.769
Atelectasis	0	1 (1.61)	-1.61 (-4.37-8.59)	NA	1.000
Pleural effusions	2 (3.23)	2 (3.23)	0 (-8.14-8.14)	NA	1.000
Pneumothorax	2 (3.23)	1 (1.61)	1.61 (-5.74-9.52)	NA	1.000
**Cardiac complications (total)**	1 (1.61)	1 (1.61)	0 (-7.1-7.1)	NA	1.000
Myocardial arrhythmia	1 (1.61)	1 (1.61)	0 (-7.1-7.1)	NA	1.000
**Gastrointestinal complications (total)**	2 (3.23)	2 (3.23)	0 (-8.14-8.14)	NA	1.000
Anastomotic leak	0	1 (1.61)	-1.61 (-4.37-8.59)	NA	1.000
Intestinal obstruction	0	1 (1.61)	-1.61 (-4.37-8.59)	NA	1.000
Delayed gastric emptying	2 (3.23)	0	3.23 (-3.06-11.02)	NA	0.496
**Other complications**					
Bleeding	2 (3.23)	0	3.23 (-3.06-11.02)	NA	0.496
Urinary tract infection	1 (1.61)	0	1.61 (-4.37-8.59)	NA	1.000
Wound infection /Fat necrosis	1 (1.61)	1 (1.61)	0 (-7.1-7.1)	NA	1.000
Unilateral RLN	4 (6.45)	0	6.45 (-0.57-15.45)	NA	0.119
**Clavien-Dindo grading system**					
I	2 (3.23)	3 (4.84)	-1.61 (-6.81-10.38)	NA	1.000
II	12 (19.35)	7 (11.29)	8.06 (-4.87-20.9)	1.554	0.213
IIIa	4 (6.45)	5 (8.06)	-1.61 (-8.48-11.85)	NA	1.000
IIIb	2 (3.23)	1 (1.61)	1.61 (-5.74-9.52)	NA	1.000
**Unscheduled readmission within 60 days**	0	0	NA	NA	NA
**In-hospital mortality**	0	0	NA	NA	NA
**90 days mortality**	0	0	NA	NA	NA

N, number; S, surgery; NAC, neoadjuvant chemotherapy; CI, confidence interval; NA, Not Available; RLN, recurrent laryngeal nerve.

## Discussion

It is challenging to adapt esophagectomy to FTS. The most controversial part of FTS for EC is the resumption of early oral feeding in cases of anastomosis leakage and aspiration pneumonia (6). Ten years have passed since Dr Li first attempted to resume oral feeding in patients on POD1. Bohle et al. (12) reported that NAC was a risk factor for anastomotic leakage. Due to the risk of complications, initially, we could only dare to perform the “non-tube no fasting” FTS in primary surgery patients. Step by step, we have tried to combine it with neoadjuvant treatment. Most resectable EC patients need to receive preoperative treatment. If this approach cannot be combined with comprehensive treatment, then “non-tube no fasting” FTS is futile. The trial conducted by Cunningham et al. (13) reported no increase in complications when using NAC and FTS. Nomoto et al. reported NAC was related to a poorer preoperative condition, however it did not worsen the short-term outcomes (14). Based on their results, we tried the combination of NAC and FTS, and we found that our “non-tube no fasting” FTS approach could be extended; therefore, we added NAC with caution. As shown in **Figure 1**, we found that the number of patients undergoing the combination of “non-tube no fasting” FTS and NAC has increased year over year. Finally, in this study, we demonstrated that the total number of postoperative complications (p=0.425) did not increase in the combined patients. From 2014-2017, NAC was not an exclusion criterion for “non-tube no fasting” FTS. After 2017, the most of the patients received NAC without consideration of FTS.

In the present study, we attempt to summarize and demonstrate the safety of NAC in combination with “non-tube no fasting” fast-track surgery. Our study showed that the combination of NAC with “no tube no fasting” fast-track surgery after McKeown MIE did not increase the incidence of anastomotic leakage (the difference rate was -1.61% (95% CI -5.3% to 8.59%)) or pneumonia (the difference rate was 1.61% (95% CI -9.79% to 13.07%)). The results of this study were consistent with the results of our previous study, although only 31.1% (87/280) of the patients received NAC.

The other aspect was the efficacy of the NAC and “non-tube no fasting” FTS combination. A short postoperative hospital stay is one of the most important recovery outcomes and the most desirable outcome of FTS. In the current study, the NAC combined with FTS group had the same median discharge day as the FTS group (8 days, p=0.723). The length of stay was also consistent with other esophageal FTS studies (6). In our study, the discharged patients returned home to resume their leisure activities and activities of daily living. The fast recovery time may also be a benefit of MIE (8, 15). All patients resumed oral feeding on POD1 in both groups, with acceptable and equivalent rates of anastomotic leakage observed. This demonstrates the efficacy of the combination of NAC and “non-tube no fasting” FTS.

Regarding other data, the NAC combined group had a significantly longer surgical time (200 min vs. 222.5 min, p<0.001). Although 22.5 min is insignificant in our daily clinical practice, it indicates that NAC may prolong the surgical time. This result was different from the findings reported in our previous study (7). However, in the present study, the data were all from one medical team, so this might be more reflective of the increased surgical difficulty due to NAC. NAC causes tissue fibrosis, inflammation and tissue edema (12, 16), all of which might contribute to the prolonged surgical time. In the NAC combined group, more lymph nodes were harvested during the operation (median 33 vs. 20, p<0.001). As the pCR rate of NAC was approximately 10% (17), more lymph nodes tended to shrink rather than disappear. This may make lymph node dissection easier and allow for more lymph nodes to be harvested. Additionally, the ease of decision making may explain why the RLN injury rate in the combined group was significantly lower than that in the primary surgery group, 4/62 versus 0/62, respectively, and the difference rate was 6.45% (95% CI -0.57% to 15.45%).

In the present study, we demonstrated that NAC combined with “non-tube no fasting” FTS was equal to “non-tube no fasting” FTS in terms of the incidence of pulmonary complications, and the rates of postoperative complications, unscheduled readmission, hospital mortality and 90-day mortality were not affected. Taken together, our results showed that the NAC combined with “non-tube no fasting” FTS is safe and does not affect the hospital discharge day, as indicated by our previous RCT with little NAC data.

### Limitations

As a single-center retrospective study, our study could not avoid natural biases. Moreover, in the current study, although PSM was adopted, some differences may have led to selection bias; for example, patients with a better performance status were more likely to receive preoperative treatment. Second, this study was performed in the highest-incidence EC area worldwide in a high-volume cancer hospital in Henan Province. A total of 997 esophagectomy procedures were performed for esophageal cancer during 2015 in our department, so the learning curve and surgical experience may be quite different from those of a low-volume center in a low-incidence area. Further exploration is needed to determine whether this approach is truly suitable for centers with limited experience. Third, this study used MIE hand-sewn cervical anastomosis. We did not know if the mechanical anastomosis also work? Fourth, similar to Japan, we were more likely to adopt NAC rather than NACR, so the number of NACRs was too limited to draw any conclusions. Finally, the total number of patients was limited. Therefore, we excluded NACR patients. This topic still needs to be addressed in the future.

## Data availability statement

The raw data supporting the conclusions of this article will be made available by the authors, without undue reservation. Requests to access these datasets should be directed to Jing Ding, dingjing201305@163.com.

## Ethics statement

The studies involving human participants were reviewed and approved by Ethics Review Committee of the Affiliated Cancer Hospital of ZhengZhou University/Henan Cancer Hospital (number 2016ct081). The patients/participants provided their written informed consent to participate in this study.

## Author contributions

YZ and WX designed the study. YL, XL, HS, ZW and YZ performed the surgical treatment and fast track surgery. WH and SL analysed the data, and YZ and WH wrote the manuscript. All authors contributed to the article and approved the submitted version.

## Funding

This work was supported by National Natural Science Foundation of China, NSFC (grant numbers 82002521); Natural Science Foundation of Henan Province (grant numbers 202300410389), China; Henan Anti-Cancer Association Youth Talent Project (grant number 2019HYTP018, 2019), China; Wu Jieping Medical Foundation (CN) (grant numbers 320.6750.2020-15-1), China; Henan Province health science and technology innovation outstanding young talents training project (YXKC2021029), China; Henan Province Medical Science and Technology Key Projects Co-constructed by the Ministry of health (SBGJ202102059), China.

## Acknowledgments

We acknowledged Yanan Qi, a statistician, to help us for the statistics. Department of Statistics, LinkDoc Technology Co., Ltd., Beijing, China.

## Conflict of interest

The authors declare that the research was conducted in the absence of any commercial or financial relationships that could be construed as a potential conflict of interest.

## Publisher’s note

All claims expressed in this article are solely those of the authors and do not necessarily represent those of their affiliated organizations, or those of the publisher, the editors and the reviewers. Any product that may be evaluated in this article, or claim that may be made by its manufacturer, is not guaranteed or endorsed by the publisher.
